# Optimal duration of progesterone before cryopreserved embryo transfer in hormone replacement therapy cycles: A prospective pilot study

**DOI:** 10.1097/MD.0000000000040864

**Published:** 2024-12-06

**Authors:** Lijuan Zhao, Liu Liu, Yongdong Dai, Feng Zhou, Chao Li, Xiaoxiao Hu, Jing Li, Yanling Zhang, Songying Zhang

**Affiliations:** a Department of Obstetrics and Gynecology, Assisted Reproduction Unit, Sir Run Run Shaw Hospital, School of Medicine, Zhejiang University, Hangzhou, China; b Zhejiang Provincial Clinical Research Center for Reproductive Health and Disease, Hangzhou, China; c Zhejiang Key Laboratory of Precise Protection and Promotion of Fertility, Hangzhou, China.

**Keywords:** frozen embryo transfer, hormone replacement therapy cycle, progesterone

## Abstract

This prospective pilot cohort study aimed to ascertain the optimal duration of progesterone supplementation prior to frozen embryo transfer (FET) in women undergoing hormone replacement therapy (HRT) cycles. A total of 127 participants were enrolled and divided into 2 cohorts. The first cohort, comprising of 39 women, was used to determine the peak period of endometrial receptivity. These participants underwent serial assessments of integrin alphavbeta3, homeobox gene A10, and leukemia inhibitory expression levels from days 3 to 7 (P + 3 to P + 7) during the mock HRT cycles. The second cohort included 88 women who embarked on their inaugural HRT-FET cycle and were monitored for pregnancy outcomes after the transfer of D3 embryos after 3 or 4 days of progesterone administration. The results indicated a significant enhancement in biochemical pregnancy and embryo implantation rates in the P + 3 group (87.18% and 74.36%, respectively) compared to those in the P + 4 group (68.42% and 65.79%; *P* = .047 and *P* = .044, respectively). These data suggest that a 3-day progesterone regimen prior to D3 embryo transfer may be more beneficial. Notably, the maximal expression of endometrial receptivity markers was observed on day P + 6 within the HRT cycles, which is consistent with previous research that correlated day 6 embryos with peak endometrial receptivity. Therefore, a 3-day progesterone lead-in may enhance synchrony between D3 embryos and the endometrium. Our findings suggest that initiating D3 embryo transfer after 3 days of progesterone administration may optimize embryo-endometrial synchronization, thereby potentially enhancing clinical outcomes in FET cycles.

## 1. Introduction

Successful embryo implantation is a complex process contingent upon the intricate interplay between a receptive endometrium and a viable embryo. The window of implantation (WOI), a critical phase during the luteal period, is typically restricted to a narrow timeframe and generally does not exceeding 2 days in duration.^[1]^ Proposed biomarkers leveraged to identify the WOI include integrin alphavbeta3 (αvβ3), homeobox gene A10 (HOXA10), and leukemia inhibitory factor (LIF).^[2,3]^ As an adhesion molecule, αvβ3 integrin is expressed on the apex of luminal and glandular cell surfaces during the WOI,^[4]^ and altered αvβ3 integrin expression patterns have been linked to unexplained human infertility.^[5,6]^ HOXA10 is a homeobox gene that has also been identified as a potential receptivity marker, with its expression rising during the luteal phase prior to peaking during the WOI.^[7,8]^ LIF controls trophoblast function and placental vascularization, with peak endometrial LIF and LIF receptor (LIFR) expression occurring during the peri-implantation phase.^[9,10]^ Interaction among these key molecules is essential for the appropriate regulation of implantation.

Advances in our understanding of biomarkers and their roles in endometrial receptivity have markedly advanced assisted reproductive technologies (ART), especially in the context of hormone replacement therapy (HRT) cycles. Synchronizing embryo transfer with endometrial WOI is pivotal for successful ART. However, the optimal timing for embryo transfer, particularly for D3 cleavage-stage embryos, remains a challenge. The central debate revolves around the duration of progesterone exposure necessary to prime the endometrium for implantation: whether a 3-day (P + 3) or 4-day (P + 4) protocol before embryo transfer is more conducive to positive pregnancy outcomes.

The literature presents conflicting views on the ideal duration of progesterone administration in HRT cycles for D3 embryos. Some studies support the sufficiency of a shorter progesterone exposure (P + 3), suggesting that it leads to a receptive endometrium and favorable pregnancy outcomes.^[11,12]^ Conversely, other studies^[13]^ advocate for a longer, 4-day progesterone priming period, arguing it better aligns with the natural timeline of embryonic development and implantation, thus potentially enhancing implantation rates and improving pregnancy success. Interestingly, additional research has found no significant differences in the outcomes between embryos transferred on P + 3 and those transferred on P + 4, suggesting that the duration of progesterone exposure might not critically impact the success of the procedure.^[14]^ This ongoing debate highlights the need for further investigation to determine the most effective timing of progesterone administration in HRT cycles. Debate over the optimal timing of progesterone supplementation has persisted. To address this inconsistency, we systematically evaluated the effect of progesterone exposure duration on implantation and pregnancy outcomes for D3 cleavage-stage embryos in HRT cycles. By meticulously assessing endometrial receptivity markers and analyzing clinical outcomes, we aimed to identify the most effective timing for progesterone administration, thereby refining embryo transfer protocols and elevating ART success rates.

## 2. Materials and methods

### 2.1. Participants

**Figure 1.**
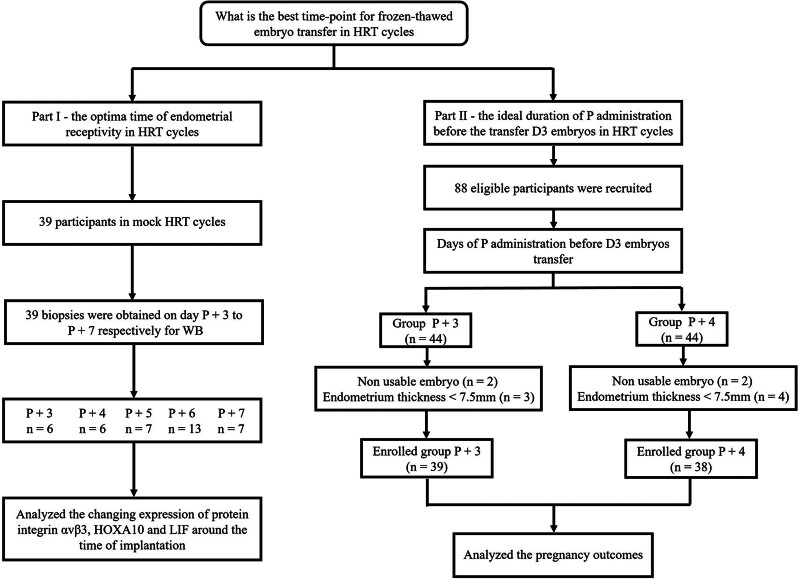
Flow diagram outlining the participant recruitment and follow-up period. αvβ3 = integrin alphavbeta3, HOXA10 = homeobox gene A10, HRT = hormone replacement therapy, LIF = leukemia inhibitory factor, P = progesterone.

The current study included 2 experimental cohorts. The first cohort consisted of 39 women undergoing mock HRT cycles recruited for daily measurements of αvβ3, HOXA10, and LIF, 3–seven days after progesterone administration. The second cohort consisted of a separate group of 88 women undergoing the first HRT cycle recruited to evaluate the pregnancy outcomes associated with the transfer of D3 embryos after either 3 or 4 days of progesterone supplementation. Eleven of these women withdrew from the study (Fig. 1). The total number of participants in this study was 116. All patients underwent in vitro fertilization-frozen embryo transfer treatment at the Reproductive Center of Sir Run Shaw Hospital between January 2015 and February 2020. The participant inclusion criteria were as follows: 25 to 35 years old; undergoing the first frozen embryo transfer (FET) following in vitro fertilization treatment; regular 26-35 day menstrual cycle; regular karyotype for both partners; and at least 2 high-quality D3 embryos obtained after thawing. Participants were excluded from this study if they had endometriosis, hydrosalpinx, or any other uterine pathology; had a prior history of cancer or other major diseases; or had undergone steroid hormone treatment within 3 months prior to recruitment.

### 2.2 Hormonal replacement protocol and endometrial biopsy

In total, 39 patients were recruited for the first study cohort, all of whom underwent a mock HRT cycle as per a previously published protocol.^[15]^ On the third day of their menstrual cycle, patients began taking oral estradiol valerate (6 mg/day; Progynova, Schering, Germany) without any GnRH analog for 7 days, after which the dose was increased to 8 mg/day for 7 to 10 days. When endometrial thickness was ≥ 8 mm, patients were intramuscularly injected with progesterone (80 mg/day) for 3 to 7 days. Endometrial biopsies were collected the morning after the final progesterone dose using a pipeline catheter (Laboratory CCD, China). After collection, samples were rinsed with normal saline and stored at −80°C before western blotting (WB) analysis.

The rationale for administering progesterone from P + 3 to P + 7 days in our study was based on the necessity of encompassing the entire spectrum of the WOI, as suggested by prior research. This interval was chosen to ensure that the critical period of endometrial receptivity was determined, allowing for a comprehensive assessment of the expression patterns of key receptivity markers. By covering this range, we aimed to identify the peak expression of these markers and determine the optimal window for embryo transfer.

### 2.3. Western blotting

Tissue samples were lysed with RIPA buffer (Beyotime, Shanghai, China) containing 1% PMSF (Beyotime), and an enhanced BCA Protein Assay kit (Beyotime) was used to determine the protein concentration in the samples. Equivalent amounts of protein were then separated via 10% SDS-PAGE and transferred onto PVDF membranes (Millipore). Blots were then blocked with 5% nonfat milk in TBS containing 0.1% Tween-20 (TBS-T) for 2 hours. Membranes were probed overnight with antibodies specific for integrin αvβ3 (1:2000, MAB1976, Millipore), HOXA10 (1:2000, ab23392, Abcam), LIF (1:1000, ab113262, Abcam), or glyceraldehyde-3-phosphate dehydrogenase (GAPDH; 1:5000, sc-25778, Abcam) at 4°C. After 3 10-minute washes with TBS-T, blots were probed with HRP-conjugated secondary goat anti-rabbit IgG (H + L; 1:5000, 111-035-003, Jackson ImmunoResearch, West Grove). After 3 additional washes, protein bands were detected via enhanced chemiluminescence (Millipore), imaged with an electrophoresis imaging system, and densitometric quantification was performed using ImageJ software. GAPDH served as the loading control.

### 2.4. Embryo transfer

In the second pilot cohort study, 88 participants were included. All patients underwent identical estrogen administration protocols during their HRT cycles. All patients underwent identical estrogen administration protocols during their HRT cycles. Seven of these patients were excluded from the study because their endometrial thickness failed to reach 7.5 mm, while 4 withdrew because of a lack of usable embryos. In total, 39 participants underwent D3 embryo (6–8 cell stage) FET 3 days after progesterone administration (day P + 3, approximately 72 hours from progesterone start to transfer). In contrast, 38 patients underwent D3 embryo FET after 4 days of progesterone administration (approximately 96 hours from progesterone initiation to transfer). Estrogen and progesterone treatments were maintained for 2 weeks after FET when pregnancy did not occur and for 3 months after FET when pregnancy did occur.

D3 cleavage-stage embryos were chosen for transfer based on their developmental stage, which aligns with the timing of the natural embryo transit to the uterine cavity. Transferring embryos at this stage allows for more physiologically relevant synchronization between the embryo and endometrium. Additionally, the use of D3 embryos facilitates the examination of implantation dynamics within the controlled context of HRT cycles, where the hormonal milieu has been standardized. This approach also mitigates the potential variability introduced by extending in vitro culture to the blastocyst stage, thereby enabling a focused investigation of the impact of progesterone timing on early implantation events.

### 2.5. Pregnancy outcomes

Biochemical pregnancies were defined as a serum β-hCG level > 20 IU/L at 12 days post-FET, while clinical pregnancies were confirmed *via* transvaginal ultrasound-mediated detection of the gestational sac at 2 weeks following a positive hCG test. Detection of a fetal heartbeat was defined as ongoing pregnancy at 5 to 7 weeks post-transfer. Implantation rate was defined as the number of gestational sacs visible on ultrasound divided by the total number of embryos transferred. Pregnancies were monitored during delivery.

### 2.6. Ethics

The present study was approved by the Reproductive Medical Ethics Committee of Sir Run Run Shaw Hospital, College of Medicine, Zhejiang University and was registered with the clinical trial registration number ChiCTR1900027941. Written informed consent was obtained from all patients.

### 2.7. Statistical analysis

GraphPad Prism 3.0 (GraphPad Software, San Diego) and SPSS v.19.0 (IBM Corp, Armonk) were used for all statistical analyses. Data were compared with Student’s *t* tests and chi-squared tests as found appropriate. *P* < .05 was the significance threshold for this study.

## 3. Results

### 3.1. Demographics

In total, 39 women were included in the first portion of the study. The demographic and reproductive characteristics of these women, including age, body mass index, prior pregnancies, parity, cause and duration of infertility, and basal hormone levels, are compiled in Table 1. Endometrial biopsies were collected on days P + 3, P + 4, P + 5, P + 6, and P + 7 from 6, 6, 7, 13, and 7 women, respectively.

**Table 1:** Participants’ characteristics and causes of infertility in the first part of study group.

Parameter	N = 39
Age (yr)	30.68 (4.62)
Duration of infertility (yr)	3.77 (2.61)
Body mass index (kg/m^2^)	20.73 (2.22)
Gravidity	1.11 (1.43)
Basal serum FSH (IU/L)	5.89 (2.23)
Basal serum LH (IU/L)	3.53 (1.71)
Basal serum E_2_ (pg/mL)	36.89 (41.15)
Male factor	12 (30.8%)
Tubal factor	27 (69.2%)

Values are mean (±SD) or number (%).

E2 = estradiol, FSH = follicle-stimulating hormone, LH = luteinizing hormone.

### 3.2. Integrin αvβ3, HOXA10, and LIF protein level dynamics from day P + 3 to day P +

**Figure 2.**
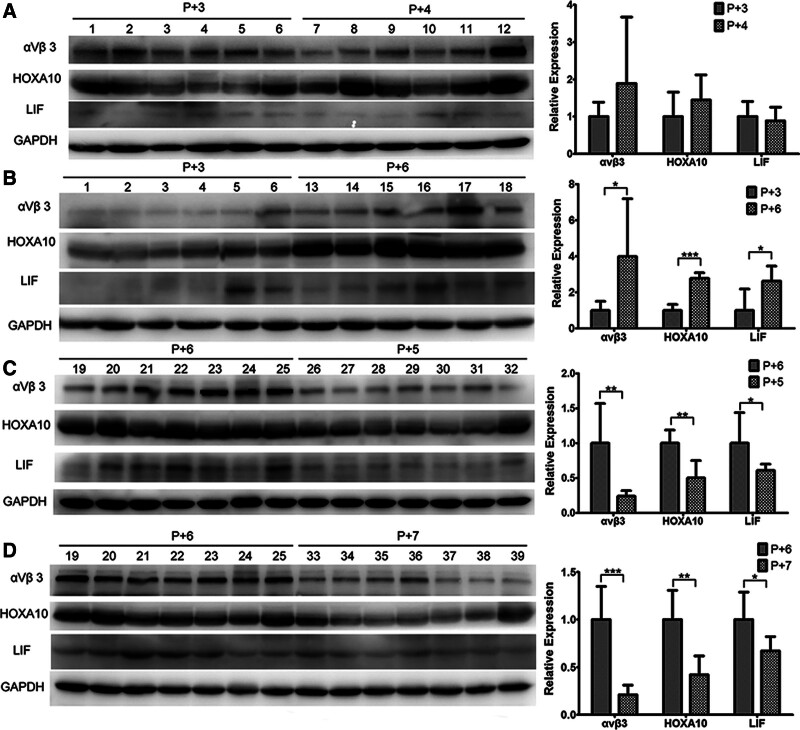
Endometrial integrin αvβ3, HOXA10, and LIF levels in women undergoing HRT cycles from days P + 3 to P + 7. Data are means ± SEM from 4 sets of experiments. (A) No differences in protein levels were detected between samples from days P + 3 and P + 4. (B) All proteins were expressed at significantly higher levels on day P + 6 relative to day P + 3. (C, D) Integrin αvβ3, HOXA10, and LIF protein levels on day P + 6 were significantly higher than on day P + 5 and P + 7. αvβ3 = integrin alphavbeta3, GAPDH = glyceraldehyde-3-phosphate dehydrogenase, HOXA10 = homeobox gene A10, HRT = hormone replacement therapy, LIF = leukemia inhibitory factor, P = progesterone.

The protein expression levels of integrin αvβ3, HOXA10, and LIF were assessed in these 39 patients, proximal to the time of implantation (Fig. 2). No significant differences were found when comparing days P + 3 and P + 4 (*P* = .2594, *P* = .2718, and *P* = .6218, respectively; Fig. 2A). However, significantly higher levels were detected on day *P* + 6 relative to day P + 3 (*P* = .0477, *P* < .0001, *P* = .0200, respectively; Fig. 2B), and the same was true on days P + 5 (*P* = .0045, *P* = .0012, *P* = .0411; Fig. 2C) and P + 7 (*P* < .0001, *P* = .0013, *P* = .0184, respectively; Fig. 2D). Our findings revealed a distinct temporal pattern in the expression of these proteins, with peak levels observed at P + 6. This peak corresponds to the mid-luteal phase, which has traditionally been associated with the highest receptivity of the endometrium. The temporal correlation between peak protein expression and the WOI supports the relevance of these markers as indicators of endometrial receptivity. Moreover, the expression profiles obtained underscore the importance of the precise timing of progesterone supplementation for optimizing endometrial preparation for embryo implantation.

Drawing on prior research^[16,17]^ which posits that the transfer of D6 embryos should align with peak endometrial receptivity, our study refines the timing of embryo transfer by pinpointing the optimal window of implantation (WOI). Our data indicate that maximal endometrial receptivity is reached on day P + 6, implying that D3 embryos should ideally be transferred after 3 days of progesterone treatment to synchronize with this window. To substantiate this hypothesis, we conducted a focused clinical trial that compared the pregnancy outcomes of D3 embryo transfers on P + 3 and P + 4, with findings supporting the adoption of our proposed timing strategy for enhanced implantation efficacy.

### 3.3. Demographic and clinical pregnancy outcome comparisons between groups

Table 2 shows patients’ demographic and clinical details in the P + 3 and P + 4 groups. There were no significant differences between these 2 groups regarding patient age, basal hormone levels, serum hormone levels on the day of FET, endometrial thickness on the day of FET, ectopic pregnancy rates or live birth rate. However, rates of biochemical pregnancy and embryo implantation in the P + 3 group (87.18%, 74.36%) were significantly (*P* = .047; *P* = .044) higher than those in the P + 4 group (68.42%, 65.79%).

**Table 2:** A comparison of the demographic parameters, clinical features and pregnancy outcomes between 2 groups of subjects.

Parameter	P + 3 (n = 39)	P + 4 (n = 38)	*P* value
Age	29.03 ± 2.57	29.23 ± 2.75	.734
BMI (kg/m^2^)	22.65 ± 2.81	22.97 ± 3.38	.796
Basal serum FSH (IU/L)	6.77 ± 1.81	6.19 ± 1.71	.212
Basal serum LH (IU/L)	5.84 ± 4.01	5.68 ± 5.07	.897
Basal serum E_2_ (pg/mL)	32.34 ± 18.52	29.40 ± 17.62	.532
ET day serum E_2_ (pg/mL)	440.92 ± 197.24	441.95 ± 186.05	.981
ET day serum P (ng/mL)	23.23 ± 6.60	24.27 ± 6.88	.502
ET day endometrial thickness (mm)	9.28 ± 1.49	8.98 ± 1.37	.364
Number of ET	1.85 ± 0.37	1.82 ± 0.39	.675
Good embryo rate (%)	88.89 (64/72)	84.29 (59/70)	.421
Biochemical pregnancy rate (%)	87.18 (34/39)	68.42 (26/38)	.047
Clinical pregnancy rate (%)	74.36 (29/39)	65.79 (25/38)	.411
Embryo implantation rate (%)	65.28 (47/72)	48.57 (34/70)	.044
Ectopic pregnancy rate (%)	3.45 (1/29)	0.00 (0/25)	.349
Live birth rate (%)	71.79 (28/39)	65.79 (25/38)	.569

Values are mean ± SD.

BMI = body mass index, E_2_ = estradiol, ET = embryo transfer, FSH = follicle-stimulating hormone, LH = luteinizing hormone, P = progesterone.

## 4. Discussion

Our evaluation of integrin αvβ3, HOXA10, and LIF expression in the endometrium at time points proximal to the WOI in patients undergoing HRT cycles revealed peak expression on day P + 6, indicating that this may represent the optimal time point for endometrial receptivity. Several metrics have been used to gauge endometrial receptivity including endometrial thickness, volume, pattern, and wave-like activity.^[18]^ in addition to traditional histological analyses,^[19]^ morphological pinopodes detection,^[15,20]^ endometrial receptivity arrays,^[21]^ molecular marker measurements,^[22]^ and the assessment of endometrial fluid aspirates.^[23,24]^ However, uncertainty persists regarding the reproducibility and clinical relevance of these different analytical approaches, and no uniform gold standard approach to evaluating endometrial receptivity has been identified to date. In the present study, we measured receptivity based on the expression of 3 well-characterized biomarkers, in a straightforward and invasive manner. In future research, our focus will be on identifying additional novel biomarkers that are predictive of implantation outcomes and can be measured in a noninvasive manner.

In this study, we observed no significant difference in clinical pregnancy or live birth rates between the P + 3 and P + 4 groups. However, the P + 3 group exhibited notably higher biochemical pregnancy and embryo implantation rates (87.18% and 74.36%, respectively) than the P + 4 group (68.42% and 65.79%, respectively), suggesting that a 3-day progesterone protocol before transferring day 3 embryos may enhance implantation success. This finding aligns with the concept that the endometrium is most receptive around the sixth day in hormone replacement therapy (HRT) cycles. A 3-day progesterone protocol may enhance the synchrony between endometrial receptivity and embryo development, potentially by closely mirroring the body’s natural hormonal rhythms. This approach ensures that the endometrium is receptive when the embryo reaches its optimal developmental stage for implantation. By maintaining heightened sensitivity of progesterone receptors and promoting gene expression patterns that are essential for successful implantation, a shorter regimen might also optimize the endometrial environment. Additionally, it may fine-tune the immune response to create an endometrial milieu that is conducive to embryo acceptance. Detailed mechanistic studies and targeted clinical trials are essential to validate these hypotheses and refine the FET protocols.

The primary limitation of our study was the sample size, which, although sufficient for statistical significance, may not reflect the full diversity of the FET patient population, necessitating a cautious approach to generalize our results. Future research should involve larger and more heterogeneous cohorts to confirm our findings in various demographic and clinical contexts. The universal applicability of our results is also subject to factors, such as clinical protocol variations, individual hormonal treatment responses, and embryo quality differences, emphasizing the need for personalized FET strategies. Comprehensive multicenter studies are needed to rigorously ascertain the broader efficacy of our timing protocol. Prospective studies with diverse patient populations and randomized controlled trials that compare different progesterone administration durations are critical for validating and refining the optimal timing for enhancing endometrial receptivity and implantation success.

## Acknowledgments

We thank Meiji (editor) for assistance with the English language. We would like to thank Jin Xiaoying and Wei Minlin for their assistance with data acquisition.

## Author contributions

**Conceptualization:** Lijuan Zhao, Songying Zhang.

**Data curation:** Feng Zhou, Chao Li, Xiaoxiao Hu.

**Formal analysis:** Lijuan Zhao, Jing Li.

**Funding acquisition**: Songying Zhang, Lijuan zhao

**Methodology:** Lijuan Zhao, Yanling Zhang.

**Resources:** Lijuan Zhao, Songying Zhang.

**Software:** Yongdong Dai.

**Supervision:** Liu Liu.

**Visualization:** Lijuan Zhao.

**Writing – original draft:** Lijuan Zhao.

**Writing – review & editing:** Liu Liu, Yongdong Dai, Feng Zhou, Chao Li, Xiaoxiao Hu, Jing Li, Yanling Zhang, Songying Zhang.
